# Testing times: the association of intolerance of uncertainty and metacognitive beliefs to test anxiety in college students

**DOI:** 10.1186/s40359-021-00710-7

**Published:** 2022-01-05

**Authors:** Christopher Huntley, Bridget Young, Catrin Tudur Smith, Vikram Jha, Peter Fisher

**Affiliations:** 1 School of Medicine, Whelan Building, Brownlow Hill, University of Liverpool, Liverpool, L69 3GB UK; 2 Health Services Research, Faculty of Health and Life Sciences, University of Liverpool, Liverpool, UK; 3 Department of Biostatistics, Institute of Translational Medicine, University of Liverpool, Liverpool, UK; 4 Medical Education and Healthcare, Bengaluru, India; 5 School of Psychological Sciences, Faculty of Health and Life Sciences, University of Liverpool, Liverpool, UK

**Keywords:** Test anxiety, Metacognition, Intolerance of uncertainty, Cross-sectional, College student

## Abstract

**Background:**

Test anxiety has a detrimental effect on test performance but current interventions for test anxiety have limited efficacy. Therefore, examination of newer psychological models of test anxiety is now required. Two transdiagnostic psychological models of emotional disorders that can account for anxiety are the intolerance of uncertainty model (IUM) and the Self-Regulatory Executive Function (S-REF) model. Intolerance of uncertainty, the stable disposition to find uncertainty distressing, is central to the IUM, while beliefs about thinking, metacognition, are central to the S-REF model. We tested for the first time the role of both intolerance of uncertainty and metacognitive beliefs in test anxiety.

**Methods:**

A cross-sectional design was used, with college students (*n* = 675) completing questionnaires assessing their test anxiety, intolerance of uncertainty, and metacognitive beliefs. Hierarchical linear regressions examined if intolerance of uncertainty and metacognitive beliefs were associated with test anxiety, after controlling for age and gender.

**Results:**

Females reported significantly more test anxiety than males. Partial correlations, controlling for gender, found intolerance of uncertainty and metacognitive beliefs were significantly and positively correlated with test anxiety. Hierarchical linear regressions found metacognitive beliefs explained an additional 13% of variance in test anxiety, after controlling for intolerance of uncertainty. When the order of entry was reversed, intolerance of uncertainty was only able to explain an additional 2% of variance, after controlling for metacognitive beliefs. In the final regression model, gender, intolerance of uncertainty and the metacognitive belief domains of ‘negative beliefs about the uncontrollability and danger of worry’ and ‘cognitive confidence’ were all significantly associated test anxiety, with ‘negative beliefs about the uncontrollability and danger of worry’ having the largest association.

**Conclusions:**

Both intolerance of uncertainty and metacognitive beliefs are linked to test anxiety, but results suggest metacognitive beliefs have more explanatory utility, providing greater support for the S-REF model. Modification of intolerance of uncertainty and metacognitive beliefs could alleviate test anxiety and help students fulfil their academic potential.

## Introduction

Testing is ubiquitous in higher education and students’ prospects for progression and graduating are dependent upon test performance. Many factors impact test performance [1]. Test anxiety is a key determinant of poorer test and academic performance [2, 3]. Test anxiety is a situation-specific form of anxiety whereby individuals appraise performance-evaluative situations as threatening [4]. Approximately 25% of college students are highly test anxious [5–7], with females reporting more severe test anxiety severity than males [2]. Highly test anxious individuals react with excessive worry about the consequences of failure, and somatic anxiety symptoms (e.g., muscle tension) in tests [4]. Worry, the cardinal feature of test anxiety [8], uses mental resources that would be better used for maximising test performance [9]. Test anxiety interferes directly with the taking of tests, and also influences students’ studying style, with test-anxious students more likely adopt a surface-learning approach [10, 11]. Given the negative effects of test anxiety on learning and test performance, understanding and treating test anxiety is essential so that students are able to fulfil their academic potential.

Many interventions for test anxiety have been evaluated [4, 12]. Unfortunately, existing psychological and educational interventions for test anxious college students have achieved only modest effects [12]. Most interventions are based upon behavioral approaches that use muscle relaxation techniques. Test anxiety might be more effectively treated by explicitly focusing on reducing worry, its key feature. Two contemporary transdiagnostic psychological models of emotional disorder that can account for worry and anxiety are the intolerance of uncertainty model (IUM) [13, 14] and the Self-Regulatory Executive Function (S-REF) model [15, 16]. Each model proposes different psychological mechanisms that result in anxiety.

The IUM consist of four components: intolerance of uncertainty, positive beliefs about worry, negative problem orientation, and cognitive avoidance [13, 14]. Intolerance of uncertainty, which has been likened to a cognitive bias in which uncertainty and ambiguity are viewed as threatening, is proposed to directly lead to worry and anxiety [13]. Positive beliefs about worry refers to beliefs that worry is a helpful coping strategy, negative problem orientation refers to individuals doubt about their problem-solving abilities, and cognitive avoidance refers to strategies aimed at avoiding or suppressing unwanted thoughts or images. Intolerance of uncertainty is the considered key component that leads both directly to worry and indirectly via positive beliefs about worry, negative problem orientation, and cognitive avoidance [13, 14, 17]. Individuals with high intolerance of uncertainty engage in worry as a means of increasing their subjective sense of control when faced with ambiguous or uncertain situations [18, 19]. Intolerance of uncertainty is associated with both worry [18] and anxiety [20–22], and experimental manipulations which increase intolerance of uncertainty results in corresponding increases in worry and anxiety [23, 24]. Meta-analyses have found intolerance of uncertainty implicated in the development and maintenance of emotional disorders [25–27]. Cognitive-behavioral therapy based upon the IUM is effective in reducing anxiety [28, 29]. The applicability of this model to test anxiety is obvious as students will be uncertain about the exact content of their examinations, the effectiveness of their test preparation, and the effectiveness of their performance. Consistent with this observation, in the only study examining intolerance of uncertainty and test anxiety, intolerance of uncertainty was linked to higher levels of test anxiety among college students [30].

The S-REF model states that a form of maladaptive self-regulation, termed the cognitive attentional syndrome (CAS), results in emotional disorder [15, 16]. The CAS has three components: (1) repetitive self-focused thinking in the form of worry, rumination, and overanalyzing, (2) attentional focus on sources of threat, and (3) unhelpful ways of coping (e.g., trying to distract oneself from one’s anxiety). This style of self-regulation has the effect of maintaining an individual’s sense of threat and distress. In test anxiety, the CAS may manifest itself as worry about the consequences of failing, active monitoring—of thoughts, emotions, and the environments—for signs of threat (e.g., ‘scanning’ their body for signs of anxiety or noticing failures in memory), and maladaptive coping efforts (e.g., test irrelevant thinking as means of distraction). The CAS primarily arises from metacognitive beliefs. Many metacognitive beliefs and processes have been implicated across anxiety disorders, with positive beliefs about usefulness of worry, negative beliefs about the uncontrollability and danger of worry, and beliefs about one’s memory, most commonly associated [31]. S-REF theory predicts ‘negative beliefs about the uncontrollability and danger of worry’ as being particularly important in emotional disorders because they elevate and perpetuate cycles of negative thinking [32]. Metacognitive beliefs are associated with test anxiety in college students, with ‘negative beliefs about the uncontrollability and danger of worry’ showing the largest associations [33, 34].

In summary, both the IUM and S-REF model appear applicable to test anxiety. Both models propose that positive beliefs about worry led to the selection of worry as a means of coping. Everyone worries from time-to-time, and worry can enhance motivation [35]. Worry becomes problematic if an individual feels they are unable to stop worrying and when it begins interfering with their life and tasks [36]. A key point of divergence between the two models are the beliefs posited that heighten and maintain worry and anxiety, namely, intolerance of uncertainty and maladaptive metacognitive beliefs. Intolerance of uncertainty and metacognitive beliefs are differentially associated with emotional disorder symptoms among college students [37]. Both intolerance of uncertainty and maladaptive metacognitive beliefs have been linked to test anxiety but no study has investigated them both together to examine if one or both sets of beliefs play an important role in test anxiety. Understanding the links intolerance of uncertainty and metacognitive beliefs have to test anxiety will help to develop a better psychological conceptualization of test anxiety; an important first step toward the development of an efficacious intervention. We derive our hypotheses from the S-REF model, which proposes that metacognitive beliefs are more important than beliefs in the cognitive domain (i.e., intolerance of uncertainty) in the genesis and maintenance of emotional disorder. Metacognitive beliefs have been shown to make a more substantive association to anxiety and depression symptomology than cognitive beliefs [38–40]. In particular, ‘negative beliefs about the uncontrollability and danger of worry’ are consistently and strongly associated with emotional disorder [31] and state and trait test anxiety [33]. We therefore hypothesise that: (1) both intolerance of uncertainty and metacognitive beliefs will each be significantly and uniquely associated with test anxiety, (2) the metacognitive belief domain of ‘negative beliefs about the uncontrollability and danger of worry’ will have largest association with test anxiety, and (3) metacognitive beliefs will be able to explain greater variance in test anxiety than intolerance of uncertainty.

## Method

### Participants and procedure

The study was granted ethical approval by the University’s Ethics Committee. A cross-sectional study design was used. A convenience sample of UK college students was recruited. The study was advertised by emails and an announcement on the university student intranet. Participants completed online questionnaires on their test anxiety, intolerance of uncertainty, and metacognitive beliefs. Questionnaire order was randomized. Participation was entirely voluntary, and participants could quit the study at any time without giving a reason. Informed consent was obtained from all participants. Those who completed the study had the opportunity to enter a prize draw for Amazon vouchers.

### Measures

#### Dependent variable: test anxiety

##### Test Anxiety Inventory [TAI; 41]

The TAI has 20-items assessing a student’s typical reactions to examinations, i.e., trait test anxiety (e.g., “Thinking about my grade in a course interferes with my work on tests”). Items are scored from 1 (“*Almost never*”) to 4 (“*Almost always*”). Total scores can range from 20–80, with higher scores indicating greater test anxiety. The TAI has good internal consistency, convergent validity, and acceptable test–retest reliability [41]. Internal consistency of the TAI in this study was excellent with a Cronbach’s alpha of 0.93.

#### Independent variables: intolerance of uncertainty and metacognitive beliefs

##### Intolerance of Uncertainty Scale-12 [IUS-12; 42]

The IUS-12 has 12 items that assess an individual’s intolerance of uncertainty (e.g., “When I am uncertain, I cannot function very well”). Items are scored from 1 (“*Not at all characteristic of me*”) to 5 (“*Entirely characteristic of me*”). Total scores range from 12 to 60, with higher scores indicating greater intolerance of uncertainty. The IUS-12 has good internal consistency, convergent validity, and acceptable test–retest reliability [42, 43] and has support for its validity and use among UK college student samples [30]. Internal consistency of the IUS-12 in this study was excellent with a Cronbach’s alpha of 0.90.

##### Metacognitions Questionnaire-30 [MCQ-30; 44]

The MCQ-30 has 30 items that assess metacognitive belief domains. It has five subscales: (1) ‘positive beliefs about worry’ (POS; e.g., “Worrying helps me to avoid problems in the future”); (2) ‘negative beliefs about uncontrollability and danger of worry’ (NEG; e.g., “I cannot ignore my worrying thoughts”); (3) ‘cognitive confidence’ (CC; e.g., “I do not trust my memory”); (4) ‘need to control thoughts’ (NC; e.g., “It is bad to think certain thoughts”); and (5) ‘cognitive self-consciousness’ (CSC; e.g., “I think a lot about my thoughts”). Items are scored from 1 (“*Do not agree*”) to 4 (“*Agree very much*”). Subscale scores range from 6 to 24, with higher scores indicating greater endorsement of metacognitive beliefs. The MCQ-30 has good internal consistency, convergent validity, and acceptable test–retest reliability [44], and is valid in UK college student samples [33]. Internal consistency of the POS, NEG, CC, NC, and CSC subscales in this study were acceptable-to-good with Cronbach’s alphas of 0.89, 0.89, 0.88, 0.73, and 0.83 respectively.

## Results

Six-hundred and seventy-five students completed the study from a total of 1,389 students who viewed the study website. The mean age of the sample was 21.03 years (*SD* = 3.11). The sample consisted of 482 (71.4%) females, 188 males (27.9%), and 5 (0.7%) did not specify a gender. The ethnic composition of the sample consisted of 552 (81.8%) who identified as White (British, Irish, or other), 40 (5.9%) as Chinese or Asian, 33 (5.9%) as from the Indian subcontinent (Indian, Pakistani, other), 16 (2.4%) as Black (British, African, Caribbean, other), 26 (3.9%) as having mixed ethnic heritage, 4 (0.6%), 2 (0.3%) of having another ethnic background, and 2 (0.3%) did not respond.

Before our main regression analyses were conducted, we explored the need to control for demographic variables. As females typically report greater test anxiety [3], worry, anxiety, and associated beliefs [45], independent *t*-tests examined for gender differences in independent (IUS-12, MCQ-30) and dependent (TAI) variables. Intercorrelations between test anxiety (TAI), intolerance of uncertainty (IUS-12), and metacognitive beliefs (MCQ-30 subscales) were examined using Pearson’s *r*. More mature students report greater test anxiety than younger college students [46] so we also examined correlations between age and study variables. Descriptive statistics and inter-correlations between trait test anxiety, intolerance of uncertainty, and metacognitive belief scores are shown in Table 1. Independent *t* tests found significant differences in scores between genders, with females scoring higher than males on test anxiety and ‘negative beliefs about uncontrollability and danger of worry’, but males scoring higher than females on ‘cognitive self-consciousness’. There were significant positive Pearson’s *r* correlations between all study variables, ranging from 0.12 to 0.60. We also examined the correlations between participant age and the study variables, finding age was only significantly correlated with ‘cognitive self-consciousness’ (*r* = 0.08, *p* = 0.034). Partial correlations found that all relationships between study variables remained significantly positively correlated when controlling for gender.

**Table 1:** Zero-order correlations (upper right quadrant) and first-order partial correlations controlling for gender (lower left quadrant), and descriptive statistics for study variables

Variable	1	2	3	4	5	6	7	Female (*n* = 482)	Male (*n* = 188)	Gender differences
								M (SD)	M (SD)	*t* test statistics
1. TAI	–	.46***	.19***	.57***	.35***	.33***	.18***	53.78 (14.71)	45.45 (13.50)	*t* = 6.74, *p* < .001
2. IUS-12	.46***	–	.33***	.60***	.36***	.57***	.35***	33.05 (10.40)	31.54 (9.28)	*t* = 1.74, *p* = .083
3. MCQ-POS	.19***	.33***	–	.28***	.16***	.32***	.31***	12.03 (4.45)	11.81 (4.62)	*t* = 0.56, *p* = .754
4. MCQ-NEG	.56***	.60***	.28***	–	.36***	.56***	.40***	15.20 (5.12)	13.13 (5.05)	*t* = 4.71, *p* < .001
5. MCQ-CC	.36***	.36***	.16***	.36***	–	.38***	.12**	11.99 (4.96)	11.69 (4.56)	*t* = 0.73, *p* = .469
6. MCQ-NC	.36***	.58***	.33***	.58***	.38***	–	.44***	11.98 (3.94)	12.49 (3.49)	*t* = − 1.58, *p* = .116
7. MCQ-CSC	.23***	.36***	.31***	.43***	.13**	.44***	–	15.28 (4.15)	16.55 (4.24)	*t* = − 3.54, *p* < .001

^**^p < .01, *** *p* < .001

*ns*, non-significant; TAI, Test Anxiety Inventory; IUS-12, Intolerance of Uncertainty Scale-12; MCQ, Metacognitions Questionnaire-30; POS, positive beliefs about worry; NEG, negative beliefs about the uncontrollability and danger of worry; CC, cognitive confidence; NC, need to control thoughts; CSC, cognitive self-consciousness

Hierarchical multiple linear regression analyses tested the hypotheses that intolerance of uncertainty and metacognitive beliefs would each explain variance in test anxiety, but that metacognitive beliefs would explain additional variance over-and-above intolerance of uncertainty. The entry method was used enter variables into the regression model, with demographics (age and gender) entered on Step 1, intolerance of uncertainty entered on Step 2, and metacognitive beliefs entered on Step 3. An additional regression analysis was then performed, in which Steps 2 and 3 were reversed, to examine if intolerance of uncertainty explained additional variance in test anxiety severity over-and-above metacognitive beliefs. Robust estimation, using bias corrected and accelerated bootstrapping techniques, which adjust for bias and skewness in the bootstrap distribution, were used (based upon 5,000 samples). Regression statistics are presented in Table 2. On Step 1, the demographic variable of age and gender explained 6% of variance in test anxiety severity. On Step 2, intolerance of uncertainty explained a further 20% of variance. Finally, on Step 3, metacognitive beliefs added 13% of variance. When Steps 2 and 3 were reversed, metacognitive beliefs explained an additional 32% of variance in test anxiety on Step 2, over age and gender, while intolerance of uncertainty then explained a further 2% of variance on Step 3. The final model accounted for 40% of variance in test anxiety severity (*R*^*2*^ = 0.40), with gender, intolerance of uncertainty, and the metacognitive belief domains of ‘negative beliefs about the uncontrollability and worry’ and ‘cognitive confidence’ all significantly and independently associated with test anxiety. Regression diagnostics revealed one multivariate outlier; this case was removed and the regression re-run, with no changes in the pattern of results found.

**Table 2:** Statistics for each step of the regression, predicting test anxiety (n = 668)

Step	Variable	Model statistics			Parameter estimates		
		*ΔR*^*2*^	*ΔF*	*p*	*β*	*b* (95% CI BCa)	*p*
1		.06	22.60	< .001			
	Age				.00	− 0.01 (− 0.37, 0.34)	.976
	Gender				− .25	− 8.33 (− 10.64, − 6.06)	< .001
2		.20	181.74	< .001			
	Age				.02	0.09 (− 0.25, 0.41)	.601
	Gender				− .22	− 7.36 (− 9.37, − 5.35)	< .001
	IUS-12				.45	0.66 (0.56, 0.75)	< .001
3		.13	29.48	< .001			
	Age				− .01	− 0.04 (− 0.35, 0.26)	.823
	Gender				− .16	− 5.12 (− 6.98, − 3.18)	< .001
	IUS-12				.18	0.27 (0.15, 0.38)	< .001
	MCQ-30-POS				.01	0.03 (− 0.20, 0.27)	.773
	MCQ-30-NEG				.42	1.21 (0.95, 1.45)	< .001
	MCQ-30-CC				.15	0.46 (0.26, 0.67)	< .001
	MCQ-30-NC				− .05	− 0.19 (− 0.53, 0.15)	.255
	MCQ-30-CSC				− .02	− 0.09 (− 0.35, 0.18)	.526

BCa, Bias-corrected and accelerated; IUS-12, Intolerance of Uncertainty Scale-12; MCQ-30, Metacognitions Questionnaire-30; POS, positive beliefs about worry; NEG, negative beliefs about the uncontrollability and danger of worry; CC, cognitive confidence; NC, need to control thoughts; CSC, cognitive self-consciousness

A test of equality of regression coefficients was used to examine our hypothesis that ‘negative beliefs about the uncontrollability and danger of worry’ would more greatly associated with test anxiety in the final model than the other belief domains. ‘Negative beliefs about uncontrollability and danger of worry’ was more greatly associated with test anxiety than intolerance of uncertainty (*F*[1, 672] = 32.45, *p* = 0.001) and ‘cognitive confidence’ (*F*[1, 672] = 35.59, *p* = 0.001).

## Discussion

This study aimed to conduct the first preliminary investigation of both the IUM and S-REF model as applied to test anxiety in college students. Specifically, the aim was to examine the relative associations of the beliefs considered central to each model, intolerance of uncertainty and metacognitive beliefs, to test anxiety. This helps identify if either, or both sets of beliefs are important to test anxiety, and will inform the future development of new and more efficacious interventions for test anxious students than currently exist.

Both intolerance of uncertainty and metacognitive beliefs were significantly positively correlated with test anxiety, even when controlling for gender. In the regression analyses, gender, intolerance of uncertainty and the metacognitive belief domains of ‘negative beliefs about the uncontrollability and danger of worry’ and ‘cognitive confidence’ were significantly associated with test anxiety. Difficulty tolerating the uncertainty of the tests and their consequences is a significant contributory factor to test anxiety and suggests that students need help in formulating and enacting effective coping strategies to deal with uncertainty. That distrust in one’s memory, or ‘cognitive confidence’ was also significantly associated with test anxiety is perhaps not surprising given that student’s often report ‘mind blanks’ in test situations [47]. Moreover, as anxiety interferes with cognitive performance (including working memory) and test taking [3, 48–50], it may lead highly test anxious individuals to doubt their mental abilities, including their memory. However, the relationship between subjective confidence in one’s memory and actual memory performance is tenuous at best [51]. Interventions for test anxiety should therefore seek to address metacognitive memory judgements. ‘Negative beliefs about uncontrollability and danger of worry’, which lock individuals into cycles of negative thinking, such as worrying about the consequences of failing a test, had the largest associations with test anxiety. If individuals believe their worry is uncontrollable, they will not attempt to cease it and reorient towards being focused on the test and will therefore suffer the consequent damage to their test performance. Therefore, modification of ‘negative beliefs about uncontrollability and danger of worry’ would be particularly important in alleviating test anxiety.

Though findings here provide support for intolerance of uncertainty, it appears that metacognitive beliefs play a greater role in test anxiety. Specifically, after controlling for intolerance of uncertainty, metacognitive beliefs explained an additional 13% of variance of test anxiety severity, but when the order was reversed, intolerance of uncertainty was only able to explain an additional 2% in variance after metacognitive beliefs were controlled. Although a significant amount of overall variance in test anxiety was explained (40%), it may be that other test anxiety specific cognitive and metacognitive beliefs and processes that are not accounted for within the IUM and S-REF model need to be identified and included within a future model of test anxiety. For example, students can make judgements of learning (JOL)—metacognitive assessments of how well they have learned information—that guides future learning [52]. JOL ratings may moderate the degree of test anxiety experienced, such that those students who judge their learning of content to be low would experience greater test anxiety due to an awareness that they may be ill prepared for the test.

This study has several limitations. First, although the sample size was adequate, there were a relatively high proportion of females (71%), which may bias findings given that females report greater test anxiety severity than males. Second, while we examined the core components of both models (i.e., intolerance of uncertainty and metacognitive beliefs), future studies should assess the models in their entirety to determine how each applies to test anxiety. Third, current levels of anxiety and mood were not assessed, and this may also result in inflated estimates. Fourth, though a cross-sectional design is appropriate for elucidating unique associations of intolerance of uncertainty and metacognitive beliefs in typical test anxiety reactions (i.e., trait test anxiety), the reliance on self-report measures within a cross-sectional design may result in inflated estimates due to common-method variance. Prospective designs that examine if intolerance and metacognitive beliefs are predictors of test anxiety severity in test situations (i.e., ‘state’ test anxiety) are now required, as are experimental studies that examine, for example, if the manipulation intolerance of uncertainty and/or metacognitive beliefs results in corresponding changes in test anxiety. Moreover, study designs that can capture within-subject process and change, as well as permitting between-group comparisons are needed. Finally, other factors have been linked to test anxiety, such as academic procrastination [53, 54], and it would be fruitful to control for these factors in future research to conduct strenuous tests of the IUM and S-REF model.

Overall, both IUM and S-REF model appear applicable to test anxiety as both intolerance of uncertainty and metacognitive beliefs are associated with test anxiety. Worry has long been considered the key feature of test anxiety [8]. However, previous psychological theories of test anxiety do not account for the initiation and persistence of worry [4]. This study provides the first tentative evidence that both intolerance of uncertainty and metacognitive beliefs are linked to test anxiety. Findings therefore suggest that the strength of test anxious students’ intolerance of uncertainty and metacognitive beliefs will need to be reduced in interventions if they are to be successful. Such interventions may take the form of traditional psychological therapy in individual and group settings but can also include modifications to curricula that may impact upon maladaptive metacognitive beliefs and intolerance of uncertainty, such incorporating practice tests, to reduce uncertainty surrounding the type and difficulty of test questions, as well as enabling students to make better judgements of their thinking processes, learning, and memory. However, it is now important to examine the causal role of intolerance of uncertainty and metacognitive beliefs before interventions based on the IUM and S-REF model are developed.

## Data Availability

Data is available upon request from the lead author (CH).
